# Hydrodynamic features of pulsed solvent extractor for separation of two metals by using the antagonistic effect of solvents

**DOI:** 10.1038/s41598-024-52027-1

**Published:** 2024-03-03

**Authors:** Mehdi Asadollahzadeh, Rezvan Torkaman

**Affiliations:** Nuclear Fuel Cycle Research School, Nuclear Science and Technology Research Institute, P.O. Box: 11365-8486, Tehran, Iran

**Keywords:** Engineering, Chemical engineering

## Abstract

Separating copper and cobalt ions is crucial due to the industry’s strategic reliance on both these elements. When the extraction process is able to significantly increase the separation factor, it becomes favorable to separate two ions. However, the presence of Cu(II) ions together with Co(II) hinders the achievement of optimum efficiency when using commonly available extractants. This study conducted the separation of the two elements using both batch and continuous methods in a pilot plant pulsed column equipped with a disc and doughnut structure. The initial step involved optimizing the key variables to maximize the separation factor using the central composite design procedure. The optimization of Cyanex272, Cyphos IL 101 concentrations, and the pH value of the aqueous phase were all adjusted to 0.024 M, 0.046 M, and 7.3, correspondingly. In the following step, the hydrodynamic characteristics and extraction performance were examined in the pulsed column of the pilot plant. The findings indicated that the presence of Cyphos IL 101 resulted in an increased separation factor and efficiency within the column. As a result, the ionic liquid enhances performance without encountering any operational issues. This additive is considered an environmentally friendly solvent and does not cause any negative impacts. Consequently, it is suggested for utilization in continuous industrial processes.

## Introduction

Cobalt and copper play a crucial role in numerous industrial sectors, including infrastructure, transportation, electronic devices, super-alloys, and catalysts used in chemical processing^1–4^. In recent years, there have been numerous difficulties encountered by the supply chain responsible for these materials^5–7^. These two elements are often found together in minerals and industrial products^8^. So, the fact that they were separated is something that was mentioned in previous studies^9–13^. Extracting both elements simultaneously from spent lithium batteries may not result in an effective recovery process due to their co-existence^14^. Many commercial extractants such as D2EHPA^15–18^, Cyanex272^19^, Cyanex 921^20^, LIX63^21^, Alamine336^22^, and TOA^23^ have been investigated in the literature for copper and cobalt extraction. The extraction procedure using these solvents has the drawback of low separation factors. Various methods have been suggested to achieve a more effective extraction process^24^. For instance, one approach is to study the combination of different extractants and their synergistic effects to achieve a higher separation factor.

A study conducted by Cheng et al. demonstrated that the separation factor for the extraction of copper from cobalt was 8.3 when using 17% Cyanex272. When 15% LIX84 was mixed with 17% Cyanex272 (A/O:5, pH = 3.5), the synergistic effect of the two extractants increased the separation factor to 11,000^25^. The variation of pH range during the extraction process demonstrates the synergistic effect. When compared to the other ions present in the waste solution, the pH range for copper ion extraction and separation is significantly altered when using a combination of Versatic 10 acid and Mextral 984H. This indicates a high separation factor for copper at low pH levels^26^. The combination of Cyanex301 and LIX984N in extracting copper demonstrated a strong synergistic effect, indicating a significant level of separation in the acidic aqueous solution with a pH of approximately 0.2^27^. An increase of pH results in a better Cu(II)/Co(II) separation with the mixture of the two extractants [0.05 M PC88A and 0.05 M Cyanex272]. A pH value of 5.2 led to the separation factor (~ 2.1)^28^. Lee and his colleagues conducted a study where they used solvent extraction techniques to separate metal ions in the leachate of spent lithium-ion batteries. They sequentially separated copper (Cu(II)) using 0.7 M Cyanex301 and 1.0 M Aliquat 336, cobalt (Co(II)) using 1.0 M ALi-SCN, while nickel (Ni(II)) remained in the final raffinate^29^. In a different investigation, a mixture of HCl dissolved in ethylene glycol (EG) and Aliquat 336 blended with kerosene were utilized to recover valuable metals like Co, Cu, and Ni from used lithium-ion batteries. Nickel was completely extracted and remained within the EG phase with a purity of 99.8%. Conversely, 62.3% of Co(II) and 18.3% of Cu(II) were dissolved, and the majority of these dissolved metal ions were transferred to the Aliquat 336 phase. Afterwards, Co(II) and Cu(II) were effectively removed from the loaded Aliquat 336 phase by using 2.0 M H_2_SO_4_^30^.

The synergistic results of LIX63, TBP, and Versatic 10 for copper separation from the synthetic leach solution showed that the solvating mechanism led to the extraction of neutral complexes such as CuCl_2_^31^. Cobalt has a high inclination to create negatively charged complexes with chloride ions, like CoCl_3_^−^ and CoCl_4_^2−^, which cannot be extracted by the system through a solvating process. Nickel, on the other hand, has a lower inclination to form complexes with chloride ions and partly generates a neutral complex, NiCl_2_. As a result, some of it was extracted. Mn, Mg, and Ca, since they do not form complexes with chloride ions and exist as positive ions, were not extracted by the system. Zinc also tends to create negatively charged complexes with chloride ions and is expected to follow a similar extraction pattern as cobalt^31^. The mixture of two extractants [LIX 84-I and LIX 622N] showed that the extraction efficiency for copper ions reaches 99.75% after three counter-current stages^32^.

In recent years, researchers have explored the practicality of environmentally friendly solvents that have minimal negative effects on the environment. Nonetheless, there is a scarcity of research papers discussing the implementation of green solvents specifically for extracting and separating copper and cobalt^33–35^. The results of bifunctional ionic liquid [A336] [CA-12] showed that the synthetic compound was very appropriate for enhancing the cobalt separation from nickel ions^36^. In another similar study, the obtained bifunctional ionic liquid from the two extractants Aliquat 336 and Cyanex272 was investigated for the copper extraction. The results showed that the extraction in the presence of ionic liquid led to increasing the separation factors (copper/cadmium ~ 8.41; iron/copper ~ 3246)^37^.

In addition to the few studies with ionic liquids, limited studies have been reported on investigate the continuous pilot plant columns for copper separation. Investigation of copper extraction in the pulsed sieve plate column has been reported in Carvalho and co-workers. The results showed that the operation conditions in the pulsed column with LIX 84-I or Acorga M5640 extractant helped to perform the extraction process with high efficiency ~ 90.5–99.5%^38,39^. The utilization of the rotating disc column with the new extractant [Cupromex-3302] for copper separation showed that the process was achieved with maximum extraction efficiency of 87.44% under the operating conditions: 375 rpm, 6.6 L/h, and 8.4 L/h for agitation speed, aqueous flow rate, and solvent organic flow rate, respectively^40^.

The successful extraction of cobalt, copper and other metals relies not only on the choice of solvents and extractants, but also on the extraction apparatus and its operational form. Altering the phase mixing by means of agitation^41–47^ or pulsing^48–50^ assists in improving the quality and efficiency of liquid–liquid extraction processes.

This paper examines the novel approach of separating copper from cobalt using a combination of green ionic liquid and Cyanex272 in a pulsed solvent extractor with disc and doughnut structure. The organic phase feedstock was prepared using batch experiments and the CCD approach, and the conditions were optimized to achieve the highest separation factor. Furthermore, the study investigates the continuous process of copper extraction and evaluates the impact of ionic liquid on the extraction and separation of copper by analyzing the hydrodynamic characteristics.

## Experimental

### Chemical agents

Experiments were performed with the preparation of two aqueous and organic phases. The two solvents Cyanex272 (Bis(2,4,4-trimethylpentyl) thiophosphinic acid) and Cyphos IL 101 (trihexyl(tetradecyl)phosphonium chloride) were used to prepare the organic phase. Kerosene was used as a diluent to reduce the viscosity of the organic phase. The aqueous solution was prepared by dissolving the salts of copper (CuSO_4_∙5H_2_O, 99%, Merck) and cobalt (CoSO_4_∙7H_2_O, 99%, Merck) at a chosen concentration of 200 mg/L in distilled water under the specific acidity. The physical properties of the used systems are presented in Table 1.

**Table 1:** Physical properties of the studied liquid system.

Materials	Physical properties	Values
Cyphos IL 101	ρ (kg/m^3^)	0.895
	µ (mPa s)	1824
Cyanex272	ρ (kg/m^3^)	0.915
	µ (mPa s)	120
Organic phase with mixture of extractants	ρ (kg/m^3^)	0.906
	µ (mPa s)	1.257
Aqueous phase with Cu(II) and Co(II) ions	ρ (kg/m^3^)	1011,
	µ (mPa s)	0.967
Organic containing Cyanex272 diluted in kerosene	σ (mN/m)	16.22
Organic containing mixture of Cyanex272 and Cyphos IL 101 diluted in kerosene	σ (mN/m)	13.16

### Methods of data collection

**Figure 1:**
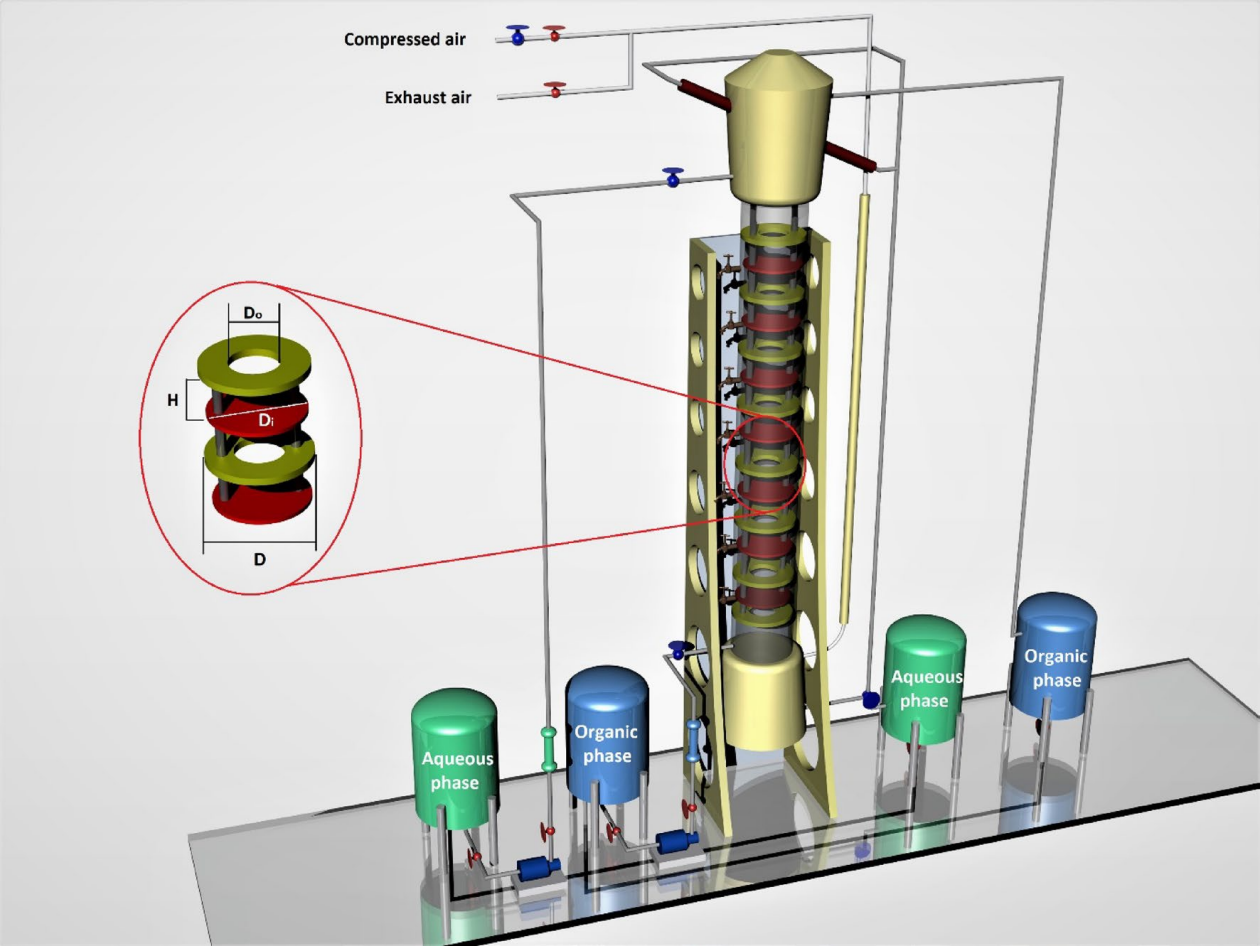
Continuous experiments in the pulsed column with disc and doughnut structure.

Two series of experiments were performed to reach the maximum percentage of copper extraction. The extraction in the batch condition was tested by an experimental design technique to investigate the concentrations of Cyanex272 extractant and Cyphos IL 101 ionic liquid, the aqueous phase acidity during the extraction and the recovery steps. The optimum point was evaluated, and it was used to prepare the feedstock for the pulsed column (ID = 76 mm; H = 740 mm). In the continuous conditions, the laboratory work was performed in the presence of organic and aqueous solvents under pulse intensity. The pulsed column (structure in Fig. 1) was initially filled with the aqueous phase, and then the streamline of the organic phase entered into the column, and two flowmeters were used to control the flow rates. A package of thirty numbers of disc and doughnut (67 mm and 36 mm, respectively) was installed inside the column to help improving the mixing.

### Analysis of data

The concentrations of copper and cobalt ions were analyzed by using spectrophotometer. Two different techniques for colorimetric following Marzenko instruction were used for the preparation of analysis solutions^51^. The extraction efficiencies for copper and cobalt ions and the separation factor are defined based on the distribution ratio with the following equations:

1 $$D = \frac{{[Cu^{2 + } \begin{array}{*{20}c} {} \\ \end{array} or\begin{array}{*{20}c} {\begin{array}{*{20}c} {} \\ \end{array} Co^{2 + } ]_{org} } \\ \end{array} }}{{[Cu^{2 + } \begin{array}{*{20}c} {} \\ \end{array} or\begin{array}{*{20}c} {\begin{array}{*{20}c} {} \\ \end{array} Co^{2 + } ]_{aq} } \\ \end{array} }}$$

2 $$\% E = \frac{D}{{D + \frac{{V_{aq} }}{{V_{org} }}}} \times 100$$

3 $$SF = \frac{{D_{Cu} }}{{D_{Co} }}$$

The taking photos were applied from four significant heights of the active section for monitoring droplet size and determining the Sauter mean drop diameter (d_32_). This technique has been reported in several studies of extraction columns^52^. The displacement method of the organic phase due to the shutdown step and the disconnection of streamlines is utilized to measure the holdup of the dispersed phase (φ), which has been used as the primary method in the previous studies^53–55^. The slip velocity (V_s_) is defined by the relative movement between both phases for the countercurrent flow, which is defined as follows:

4 $$V_{s} = \frac{{V_{d} }}{\varphi } + \frac{{V_{c} }}{{\left( {1 - \varphi } \right)}}$$

## Results and discussion

### Results of batch experiments

Batch-scale experiments were performed by combining a series of experiments with the variation in the initial aqueous pH, presence of ionic liquid Cyphos IL 101, Cyanex272 extractant by the following of Table 2.

**Table 2:** Definition of independent variables and their levels in the batch experiments.

Independent variables	Symbols	− α	− 1	0	+ 1	+ α
Concentration of cyphos IL 101/M	X_1_	0.01	0.02	0.03	0.04	0.05
Concentration of cyanex272/M	X_2_	0.01	0.02	0.03	0.04	0.05
pH of aqueous solution	X_3_	5.5	5.9	6.5	7.1	7.5

**Figure 2:**
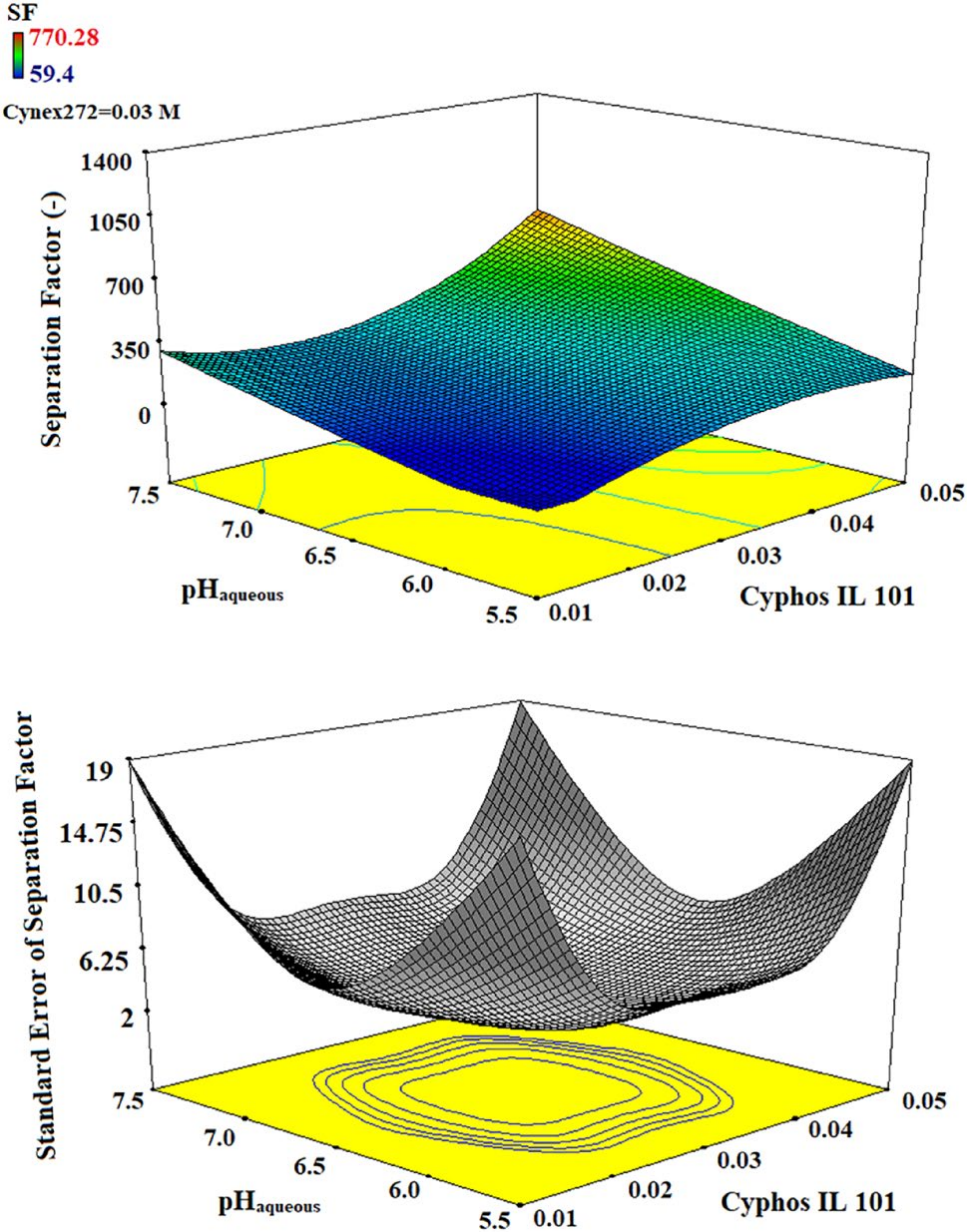
3D plots of separation factor and standard error by the variation in the concentration of Cyphos IL 101 and the initial aqueous pH.

**Figure 3:**
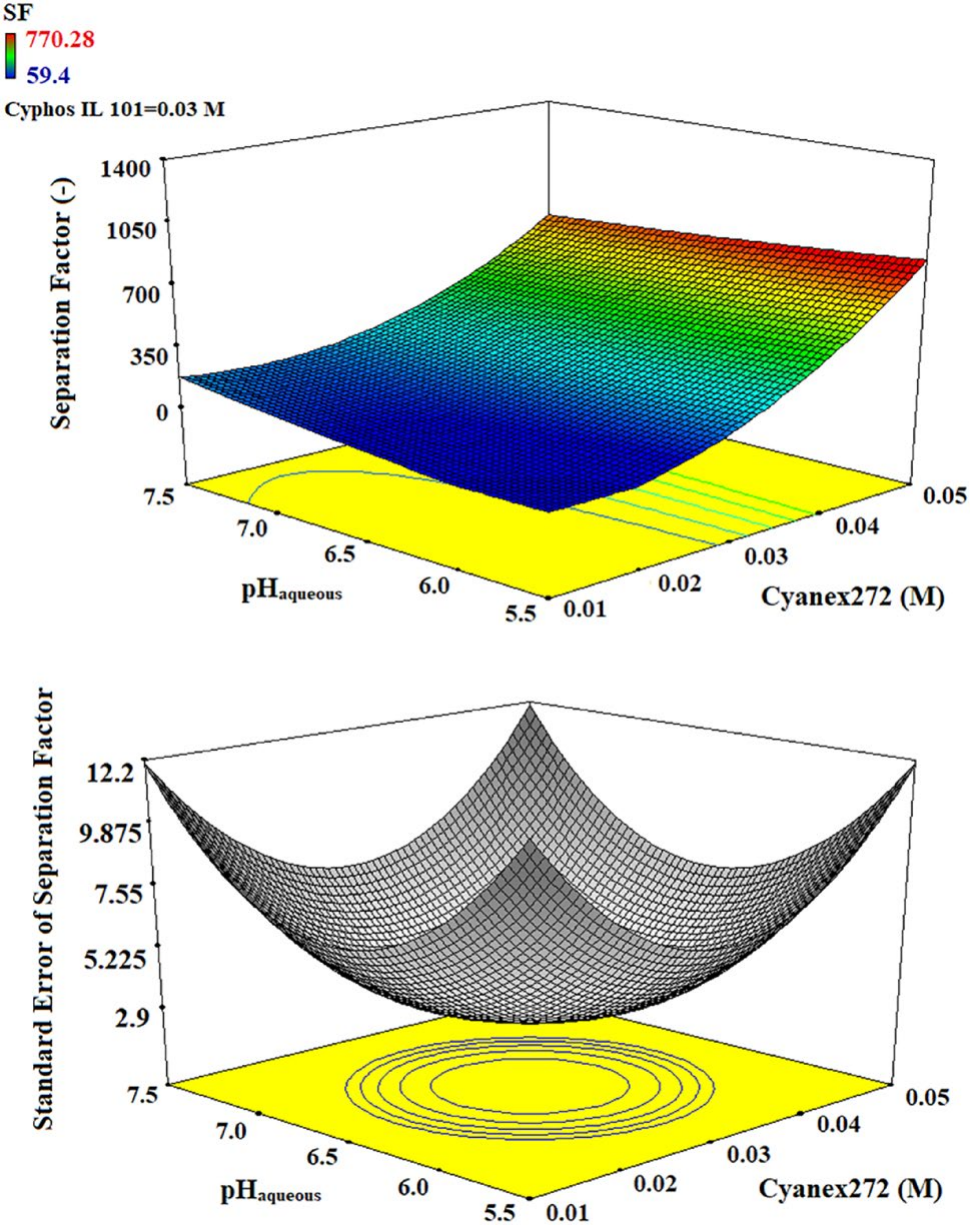
3D plots of separation factor and standard error by the variation in the concentration of Cyanex272 and the initial aqueous pH.

**Figure 4:**
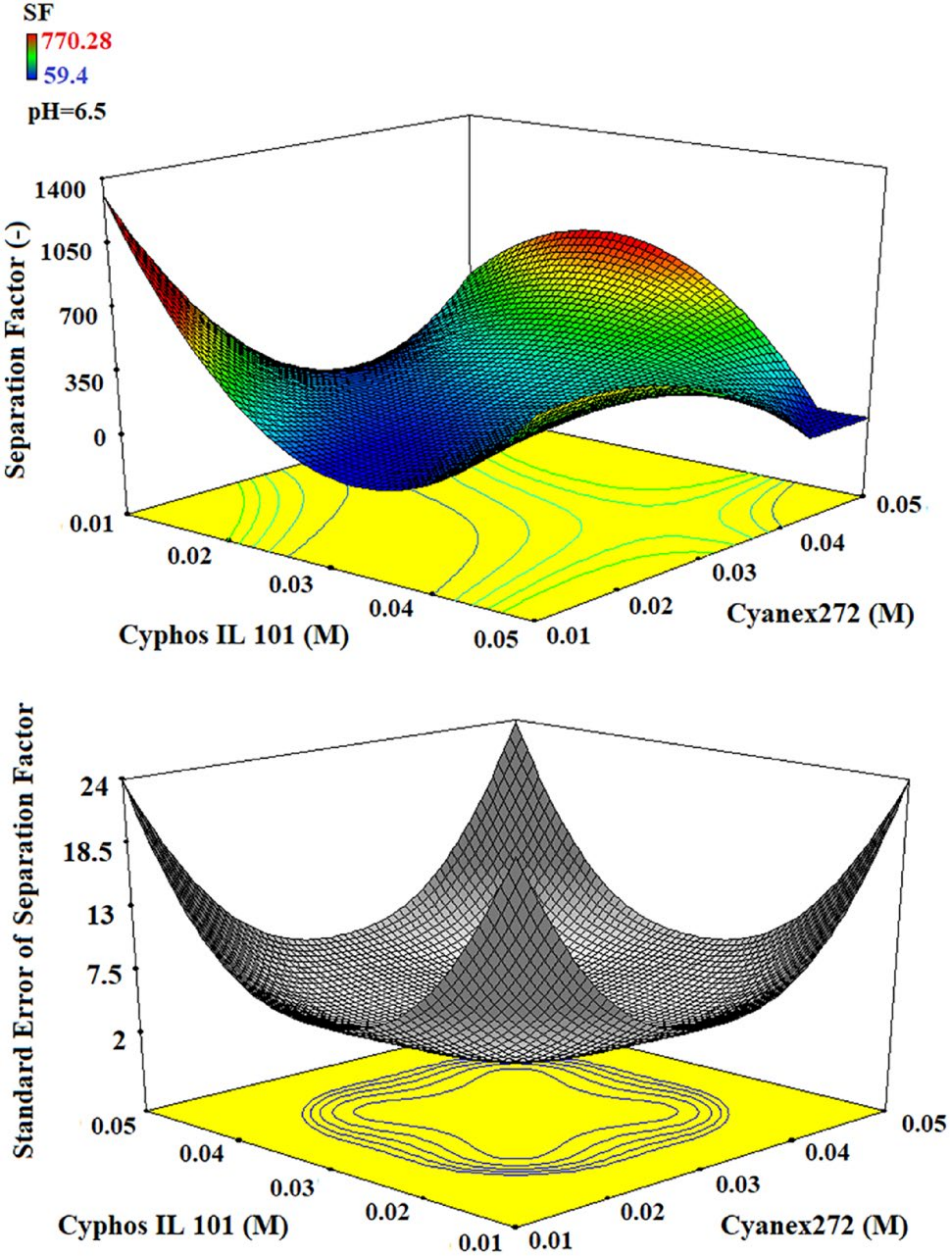
3D plots of separation factor and standard error by the variation in the concentration of Cyphos IL 101 and Cyanex272.

The required time in all experiments was one hour to ensure for reaching of equilibrium conditions. The three-dimensional diagrams derived from the variations of these parameters based on the separation factor are described in Figs. 2, 3, and 4. The results in Fig. 2 showed that the separation factor slowly increases with the increase in the initial aqueous pH and the concentration of the ionic liquid. This effect is due to the rise in the copper extraction, but the cobalt extraction continues with a very low variation, causing the increment in the difference between their distribution coefficients and the higher separation factor.

The results in Fig. 3 illustrated that, when the concentration of ionic liquid Cyphos IL 101 is constant (0.03 M), the increase in pH value due to the decrease of hydrogen ions led to the higher tendency for complex formation and releasing hydrogen ions by using Cyanex272 as the extractant. This condition exhibited a greater separation factor. When the Cyanx272 concentration increases from a low value (0.01 M) to higher values (0.05 M), it enhances the formation of the complex and is accompanied by an increase in the separation factor. This outcome suggests that although higher copper extraction is preferred, the process remains sluggish for cobalt ions. This is due to the fact that cobalt ions require higher concentrations of Cyanex272 in order to achieve a more efficient extraction rate. Devi’s research indicates that a concentration of 0.06 M is optimal for achieving maximum cobalt extraction using the Cyanex272 extractant^56^. On the other hand, Park’s study suggests that a concentration of 0.2 M is deemed suitable^57^.

The results in Fig. 4 depicted that an increase in the separation factors is obtained when the Cyanex272 concentration increases to more than 0.03 M, and in this case, the increase in ionic liquid concentration helps to reach the maximal separation factor.

**Figure 5:**
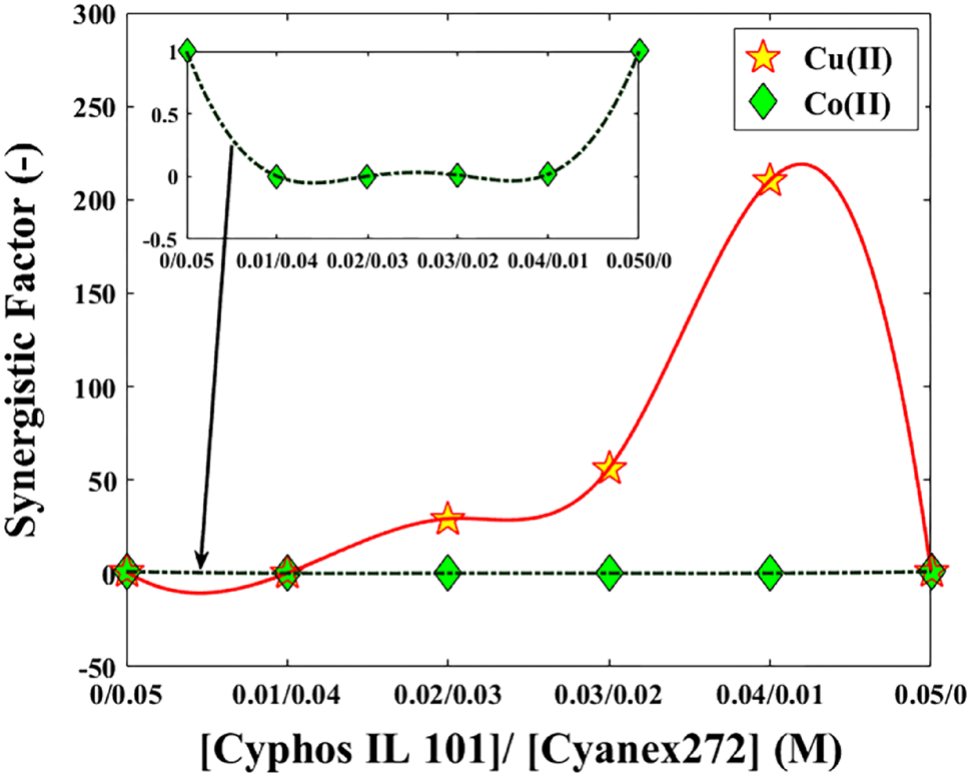
Mixture of Cyphos IL 101 and Cyanex272 with the synergistic and antagonistic effects for copper and cobalt ions.

**Figure 6:**
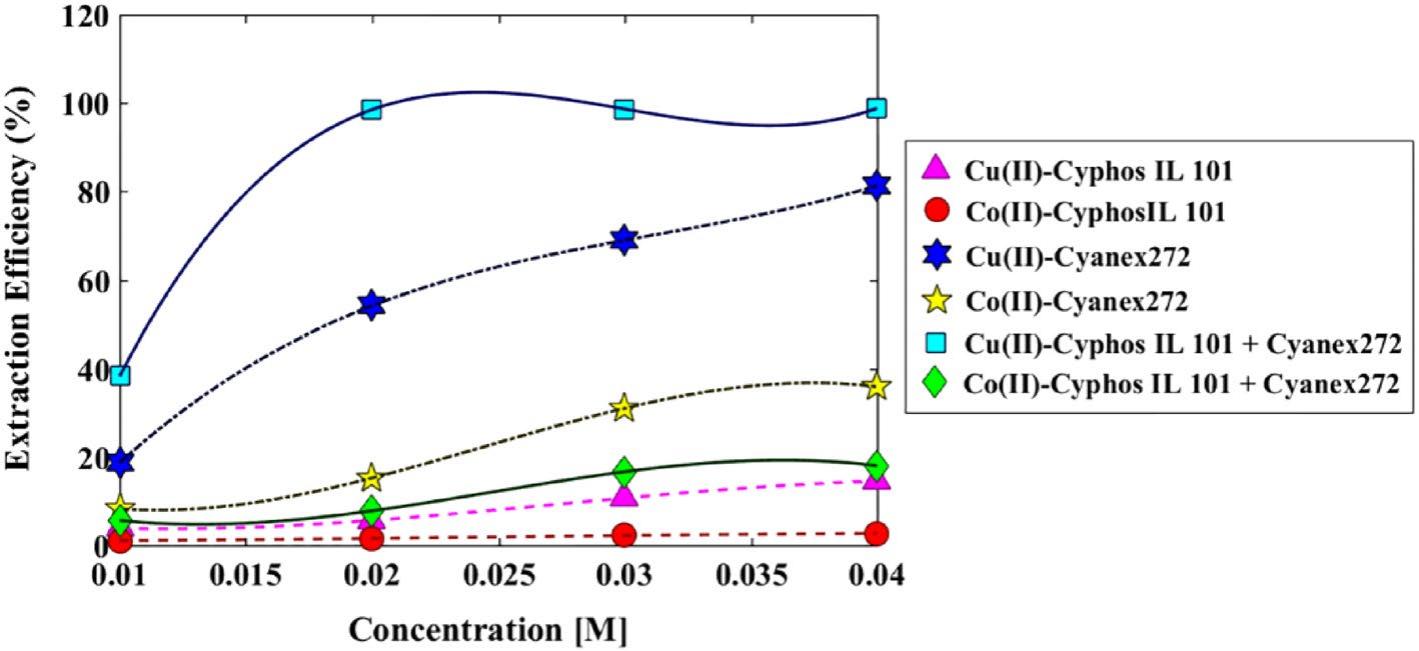
Variation in extraction efficiency for Cu(II) and Co(II) by using three organic phases from single and binary of Cyphos IL 101 and Cyanex272.

For a better description, the analysis of extraction efficiencies under different conditions with Cyanex272, Cyphos IL 101, and the mixtures from two agents is shown in Fig. 5. Also, the results of the synergistic factor (SC) (see Fig. 6) is illustrated by the definition of the following equation:

5 $$SC = \frac{{D_{mixture} }}{{D_{{Cyphos\begin{array}{*{20}c} {} \\ \end{array} IL\begin{array}{*{20}c} {} \\ \end{array} 101}} + D_{Cyanex272} }}$$

Figure 5 illustrates that when Cyphos IL 101 and Cyanex272 are combined, they enhance the synergistic factor, suggesting that utilizing this mixture is advantageous for the extraction of copper. However, the graph also indicates that the combination of these two solvents has a detrimental impact on the synergistic factor for cobalt ions, as it falls below a value of 1. Figure 6 demonstrates that the efficiency of copper ion extraction improves when both solvents are mixed, although the extraction rate remains considerably low for cobalt ions.

Thus, the results indicate that by using an ionic liquid with antagonistic effects on Co(II) ions and synergistic effects on Cu(II) ions, the separation factor can be increased. Consequently, this ionic liquid can be employed as an additive in copper separation systems, leading to reduced solvent consumption and improved performance.

To determine the optimal point, the Design-expert software was utilized to evaluate three target parameters within selected intervals, along with the maximum value for the separation factor. The findings revealed that the combination of 0.046 M Cyphos IL 101, 0.024 M Cyanex272, and pH_aqueous_ ~ 7.3 yielded the highest separation factor, which was measured at 774.14. To verify the validity of this optimal point, this combination was tested three times, with the average results demonstrating a maximum separation factor of 739.5, signifying an error of less than 4.16%. Cheng’s study revealed that employing a significant amount of extractants (17% Cyanex272. + 15% LIX84 ) leads to a separation factor value of 11,000^25^, but this approach is not cost-effective since it involves substantial expenses for extractant usage and may cause potential environmental problems. Nevertheless, this analysis achieved a remarkable separation factor of 774.14, which is noteworthy considering that previous studies utilizing ionic liquids for comparison purposes had not reported such a finding.

### Results of continuous experiments

**Figure 7:**
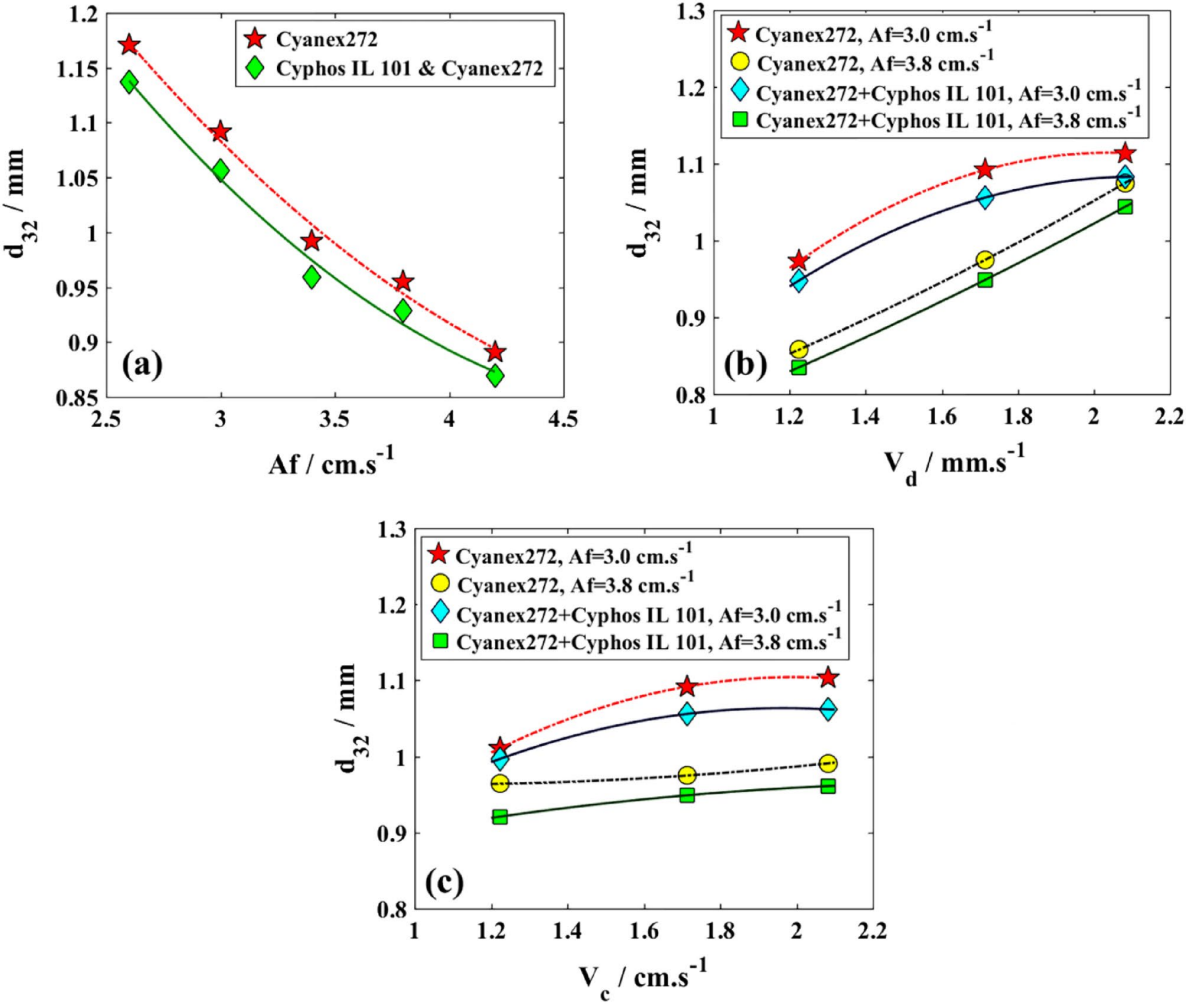
Effects of two systems for the extraction of copper and cobalt ions by the variation in the mean drop sizes; (**a**) V_d_ = V_c_ = 1.714 mm/s; (**b**) V_c_ = 1.714 mm/s; (**c**) V_d_ = 1.714 mm/s.

**Figure 8:**
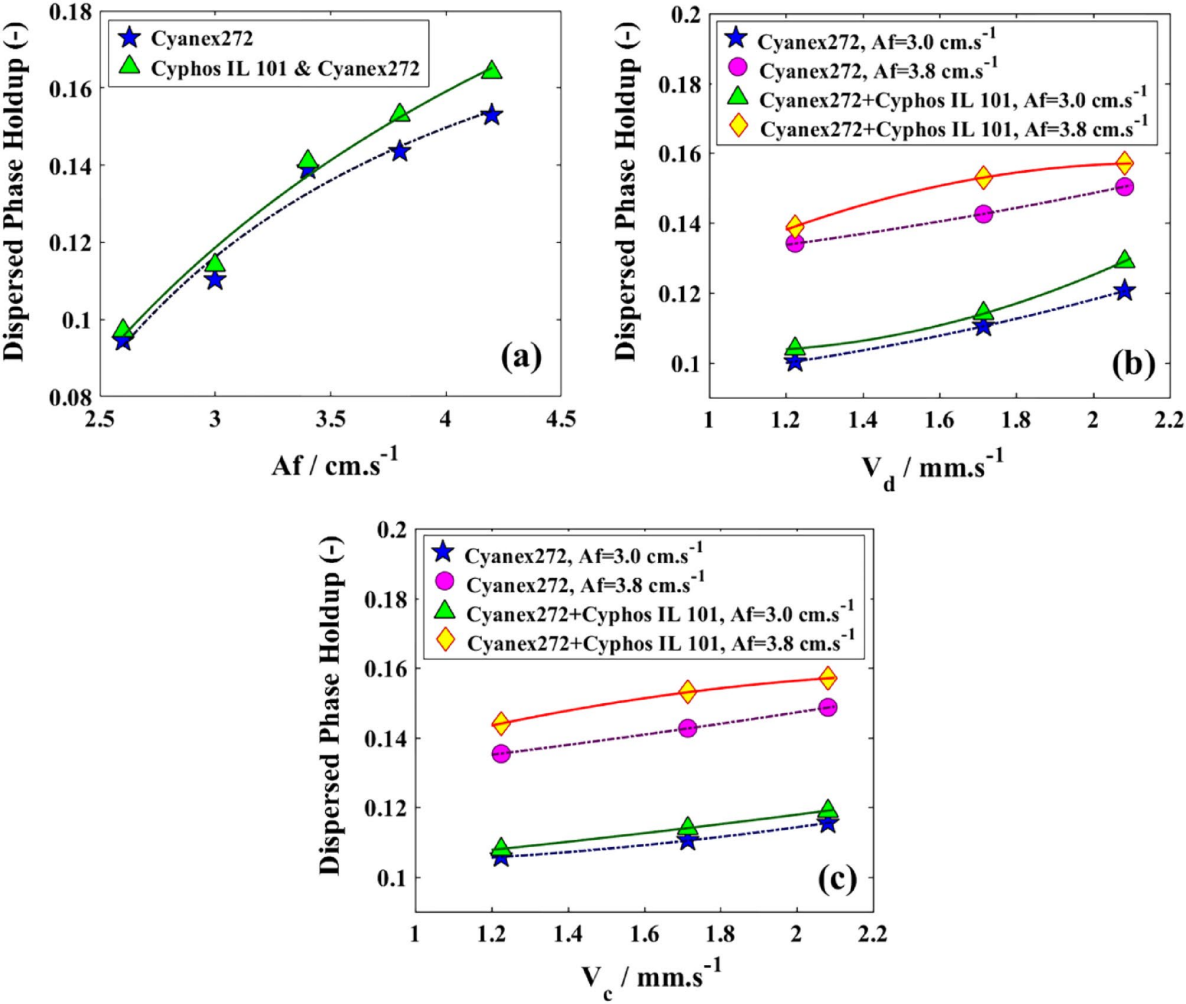
Effects of two systems for the extraction of copper and cobalt ions by the variation in the holdup of dispersed phase; (**a**) V_d_ = V_c_ = 1.714 mm/s; (**b**) V_c_ = 1.714 mm/s; (**c**) V_d_ = 1.714 mm/s.

**Figure 9:**
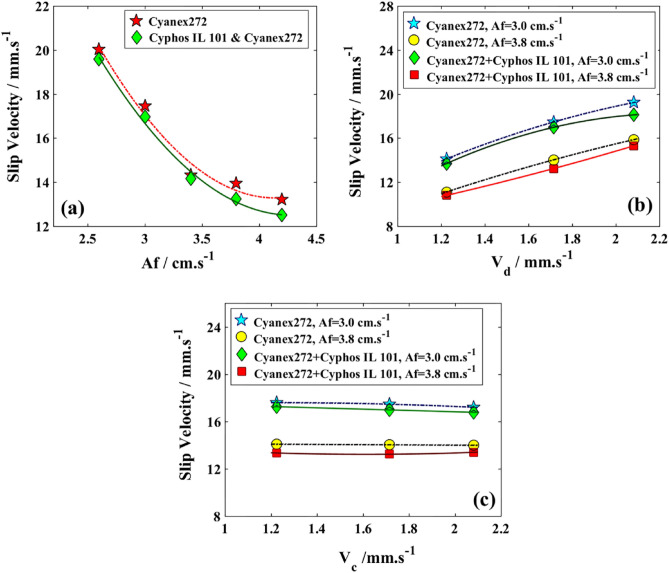
Effects of two systems for the extraction of copper and cobalt ions by the variation in the slip velocities; (**a**) V_d_ = V_c_ = 1.714 mm/s; (**b**) V_c_ = 1.714 mm/s; (**c**) V_d_ = 1.714 mm/s.

Figures 7, 8, and 9 portray the hydrodynamic characteristics of the column, demonstrating the mean droplet diameter, the changes in the holdup of the dispersed phase, and the influence of slip velocity, each depicted in separate diagrams.

The main observation in Fig. 7a is the decrease in average droplet size as the pulsation intensity increases, which is caused by the stronger shear forces that lead to droplet breakage. Additionally, the presence of the ionic liquid and lower interfacial tension result in smaller sizes for the mixture of the two organic solvents. In Fig. 7b, an increase in the organic phase’s superficial velocity leads to a higher rate of droplet coalescence and higher values for d_32_. Figure 7c shows a minimal change in the superficial velocity of an aqueous solution containing Cu(II) and Co(II) ions, indicating a lower rate of larger droplet formation. The obtained results confirm that the reduction in interfacial tension observed in physical systems leads to a decrease in droplet size, aligning with similar findings^52^.

Figure 8 illustrates the impact of using two distinct organic feeds on the holdup during Cu(II) extraction. The behavior observed in Fig. 8a suggests similarities between the pulse effects and those exhibited by physical systems^58,59^ and that further breakage rate contributes to the increase of φ, especially for a system with lower interfacial tension. The rise in V_d_ (refer to Fig. 8b) suggested that there was an increase in the number of droplets entering the column from the organic feeds, resulting in an enhancement of φ. The impact of V_c_ (refer to Fig. 8c) resembled those observed in the extensively studied system of zinc and molybdenum extraction in this column, with a minor increase in the holdup^60,61^.

Figure 9 shows the influence of increased pulsation factor and superficial velocities in the presence of ionic liquids on copper transfer. Figure 9a illustrates that slip velocity decreases with higher Af values and the presence of more holdup due to the ionic liquid leads to lower slip velocity. Figure 8b and c demonstrate how the slip velocities vary with an increase in φ and d_32_ values. The impact of superficial velocity on the organic phase is more significant than the dispersed phase holdup, which can be observed in Fig. 9b where slip velocity is enhanced even in the presence of ionic liquid. Figure 9c shows that there are no significant results observed with an increase in V_c_ for slip velocity, and a decrease is even more noticeable at lower intensities due to longer residence time for organic droplets.

**Figure 10:**
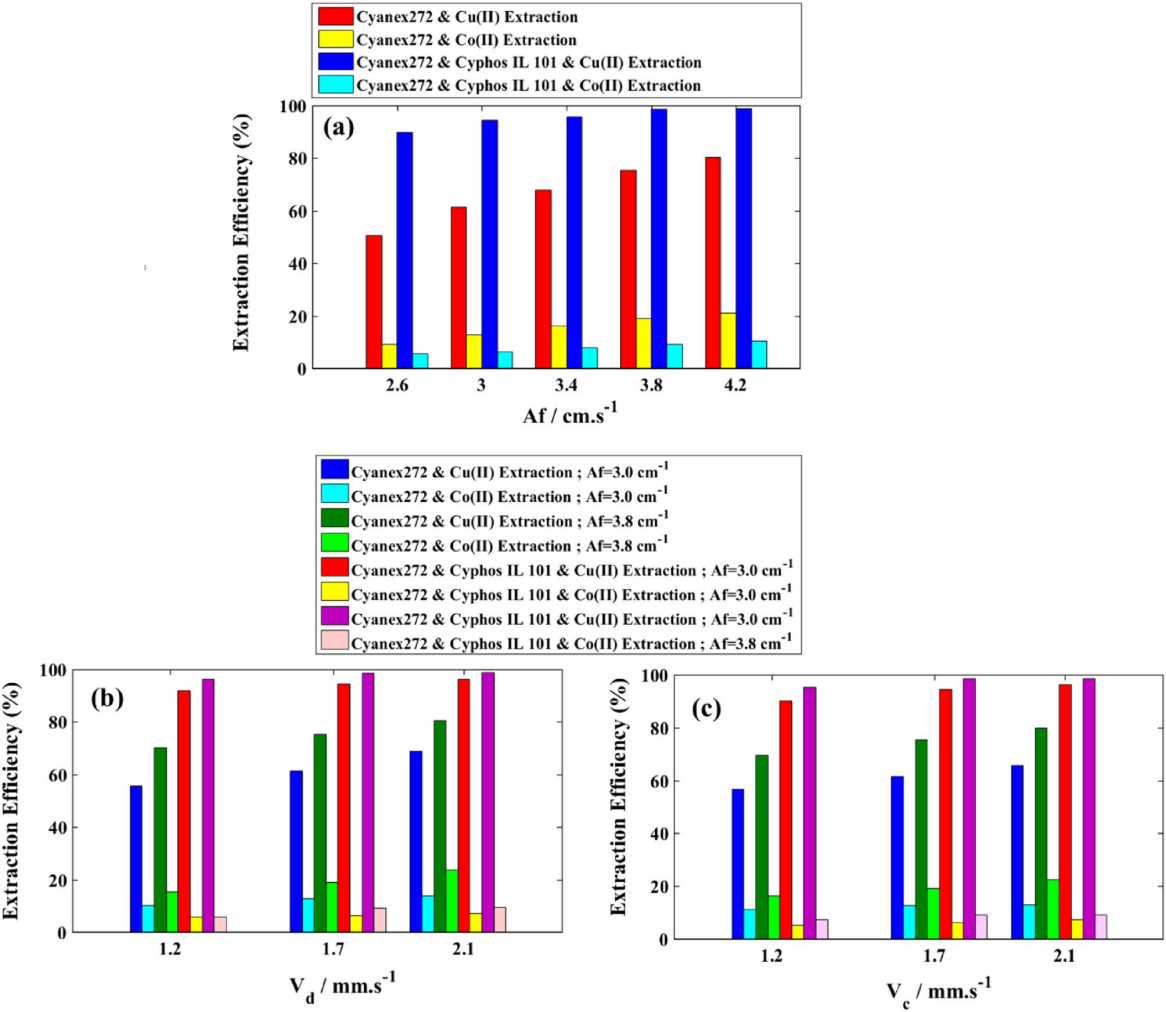
Effects of operating parameters on the extraction efficiency for cobalt and copper ions; (**a**) V_d_ = V_c_ = 1.714 mm/s; (**b**) V_c_ = 1.714 mm/s; (**c**) V_d_ = 1.714 mm/s.

The results depicting the performance of the column in extracting copper and cobalt ions, as well as their separation factor, can be observed in Fig. 10 and Table 3. The influence of pulse intensity (represented in Fig. 7a and Fig. 8a) suggests that a higher rate of droplet breakage results in a larger specific area for mass transfer. This effect ultimately improves the efficiency of copper ion extraction, increasing it from 50.45 to 80.34% when using only Cyanex272 in the organic phase, and from 89.67 to 98.88% when a mixture of Cyanex272 and Cyphos IL 101 is employed. The alterations in superficial velocities (as seen in Fig. 10b) indicate a slight increase in the extraction percentage of both elements, albeit with less impact compared to the pulse effect. This can be attributed to the heightened contact area between phases, resulting from a slower slope and ultimately leading to a higher rate of mass transfer. These patterns are similarly reflected in the data presented in Table 3, which examines the influence of operating parameters on the separation factor. In Fig. 10, the key observation is the elevated rate of copper extraction and the lower rate of cobalt ion extraction when ionic liquid is added. Consequently, the utilization of organic feed containing the ionic liquid leads to a significantly higher separation factor, increasing from 15.32 with 0.05 M Cyanex272 to 756.55 with a mixture of 0.024 M Cyanex272 and 0.046 M Cyphos IL 101. The combination of solvents in an antagonistic manner contributes to the enhanced separation factor, thereby improving the performance of the column in extracting copper ions from cobalt ions.

**Table 3:** Effects of operating parameters on the separation factor for cobalt and copper ions.

Af (cm/s)	Vd (mm/s)	Vc (mm/s)	Separation factor (D_Cu(II)_/D_Co(II)_)	
			Cyanex272	Cyanex272 + Cyphos IL 101
2.6	1.715	1.715	10.09	150.60
3	1.715	1.715	10.99	249.30
3.4	1.715	1.715	11.05	264.68
3.8	1.715	1.715	12.93	729.06
4.2	1.715	1.715	15.33	756.55
3	1.225	1.715	10.90	183.25
3	2.082	1.715	13.92	327.52
3.8	1.225	1.715	12.91	423.20
3.8	2.082	1.715	13.16	775.83
3	1.715	1.225	10.51	165.10
3	1.715	2.082	12.87	333.27
3.8	1.715	1.225	11.55	248.01
3.8	1.715	2.082	13.64	756.12

## Conclusion

This study investigated a new method of separating copper ions from cobalt ions through the antagonistic effect of an ionic liquid. The separation factor of Cu(II) from Co(II) was examined using the central composite design approach with the organic solvent Cyanex272. The addition of ionic liquid Cyphos IL 101 was used to illustrate the extraction behavior. Batch studies revealed that this ionic liquid had no impact on cobalt extraction and even had negative effects, while increasing the separation factor in leach solution systems containing copper ions. The optimal feed obtained from batch experiments was then utilized in a pulsed column with a disc and doughnut structure. The results demonstrated that the performance of the column, using Cyanex272 extract under reactive conditions to extract copper and cobalt ions, was comparable to non-reactive systems. Additionally, the inclusion of the ionic liquid yielded better performance due to its larger specific surface area for mass transfer. The high extraction efficiency of 98.88% and a separation factor of 756.55% indicated the success of the pulsed column in separating systems containing ionic liquids.

## Data Availability

The datasets used and/or analysed during the current study available from the corresponding author on reseanable request.
